# Work-related risk factors for Carpal Tunnel Syndrome among Majmaah University female touchscreen users

**DOI:** 10.12669/pjms.35.5.683

**Published:** 2019

**Authors:** Walaa Sayed Mohammad

**Affiliations:** Dr. Walaa Sayed Mohammad, PhD, Department of Physical Therapy, College of Applied Medical Sciences, Majmaah University, Majmaah, Saudi Arabia

**Keywords:** Carpal tunnel syndrome, Female, Majmaah University, Touchscreen users

## Abstract

**Objectives:**

This study aimed to explore the prevalence of carpal tunnel syndrome symptoms among female touchscreen users at Majmaah University, Saudi Arabia and to make a comparison for the wrist range of motion between probable CTS and non-CTS female touchscreen users.

**Methods:**

Two hundred and twenty-two female touchscreen users were enrolled in the present study. Among this cohort, fifty-two were academic members, 40 were employees and 130 were undergraduates. A Digital Inclinometer device was used to assess ROM of the wrist movements. A computer-based questionnaire, Phalen’s test, and Tinel’s sign were used to investigate the presence of CTS symptoms. The study was conducted between November 2018 and February 2019 at Majmaah University.

**Results:**

The prevalence of probable CTS was 34.2% among touchscreen users; the percent of probable CTS was significantly higher in undergraduates compared to other touchscreen users. There was a significant reduction in wrist flexion between the tested groups.

**Conclusion:**

Female touchscreen users at Majmaah University tended to have a high-risk for CTS. Wrist ROM measurements, particularly wrist flexion, could be a beneficial indicator for anticipating deviations in wrist posture after long-term touchscreen use. It is necessary to consider the job nature, age, BMI, and duration of using touchscreen as risk factors for CTS symptoms.

## INTRODUCTION

Nowadays, touchscreen interfaces have widely been used.^1^ This could relate to a range of their benefits, for example, easy access to information, easily communication with other, small size and mass, being connected everywhere, and so forth. In addition, touchscreen recently played an important role in the learning process as both teachers and students can easily communicate and share their knowledge. For example, the students use their smartphones for imaging information, test result queries and checking e-mails.^2^ Moreover, the academic staffs have utilised the smartphones as a method for knowledge sharing, as well as using social media applications in teaching and learning.^1^ Although of their several positive aspects, University students in Saudi Arabia are at risk of smartphone addiction. Such a phenomenon is associated with a number of negative effects on the lifestyle of users such as sleep, eating habits, energy level and finally academic achievement.^3^

Previous literature stated that smartphones being considered as a significant source of distraction for decision-based activities such as driving, classroom learning, and work-related tasks.^4^ In addition, prolonged use of touchscreen devices has been linked to a range of musculoskeletal disorders.^5^ For examples, higher musculoskeletal stresses on the neck have been related to tablet usage rather than using a desktop computer.^6^ Moreover, smartphone use may cause greater stress on the wrist compared to a keypad phone use.^7^ Additional musculoskeletal symptoms in areas supplied by the median nerve may also be related to higher usage of touchscreen devices.^8^ For instance, the median nerve changes that reported with touchscreen devices may increase the risk for narrowing of carpal tunnel^9^, increased pressure in the carpal tunnel^10^, and consequently lead to carpal tunnel syndrome (CTS).^11^ Nevertheless, such evidence is still limited with only a small number of studies assessing symptoms associated with touchscreen use.

Little is known about the prevalence and the work-related risk factors of CTS in the Saudi Arabia^12^, particularly the university members who regularly use the different kinds of touchscreen interfaces including laptop, smartphones, iPad or any touchscreen interface. Therefore, the objectives of this study will be:

- To explore the prevalence of CTS symptoms among a cohort of female touchscreen users in the University of Majmaah.
- To compare the range of motion (ROM) of the wrist in touchscreen users who suffer from CTS symptoms with the non-CTS users.
- To investigate the impact of factors such as occupation, age, predominant hand and duration of touchscreen usage on the prevalence of CTS.

## METHODS

A descriptive cross-sectional study design was used. Two hundred and twenty-two females ranging from 20-56 years were randomly chosen to participate in the current study. The number of subjects was determined a priori based on statistical power analysis to ensure type I error did not exceed 0.05 and type II error did not exceed 0.20. This analysis indicated that 211 subjects were required to find a power of 96% and level of significance of 95%. This group involved 52 academic members, 40 employees, and 130 undergraduates. Females who frequently use the touchscreen interface were able to join this study. However, females who have a history of particular medical conditions such as hypothyroidism, diabetes mellitus, rheumatoid arthritis, cervical radiculopathy, thoracic outlet syndrome, previous hand injury or surgery, and medically diagnosed CTS (by nerve conduction studies) were excluded from the present study.

Testing is essential in determining a diagnosis of CTS, so Phalen’s test and Tinel’s sign were conducted. For Phalen’s test, each participant was asked to rest her elbows on the table. Then the participant was instructed to maximally flex her wrist and hold the dorsal aspect of her hands for 60 sec. The test was considered positive when the participant experiences numbness and tingling within the test period in the median nerve distribution. For the Tinel’s sign the therapist apply light tapping along the course of the median nerve until tingling sensation is felt in the participant’s fingers. Baseline Digital Inclinometer instrument (Model 12-1057, Fabrication Enterprises, Inc., NY) was used to measure wrist ROM.

All participants were asked to fill up a self-administrated demographic questionnaire. The study was conducted between November 2018 and February 2019 at Majmaah University. The participant was asked to specify the wrist/hand pain experienced in a typical day within the past two weeks. The criteria for diagnosing the probable CTS was as the following: 1. Pain, paraesthesia, numbness, tingling, burning or decreased sensitivity in at least one of 1st, 2nd or 3rd digit, palm or wrist pain. 2. A positive Phalen’s test and/or positive Tinel’s sign. After the familiarisation session for ROM test procedure, a rest period of one-minute was given and then the ROM test was measured in random order. Before commencing the data collection session, all participants read and signed a written informed consent statement approved by the Ethical Review Committee of Majmaah University.

### Statistical analysis

Statistical Package for Social Sciences (version 24.0 for Windows; SPSS Inc., Chicago, IL) was used to analyse the data. The level of significance was set at *p* < 0.05 for all statistical tests. Females with probable CTS and non-CTS were compared on each independent variable using the chi-square test, or t-test, as appropriate. Demographic (body mass index ‘BMI’, age, predominant hand) and occupational factors (job, hours of using touchscreen /day, years of using touchscreen, and used finger) were considered as independent variables. Additionally, a two-way multivariate analysis of variance (MANOVA) was used in order to compare the wrist range of motion in both probable CTS and non-CTS groups.

## RESULTS

**Fig.1:**
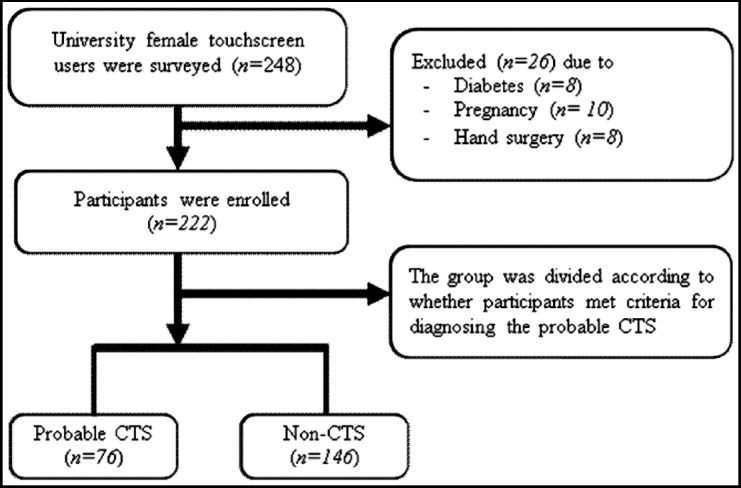
Study Flowchart of the female touchscreen users.

Based on CTS criteria, 76 participants (34.2%) had probable CTS. The prevalence of probable CTS was extraordinarily high in undergraduate females (47.4%). Table-I shows the demographic criteria of the tested groups. For this study, 248 University female touchscreen users were surveyed (Fig.1), 26 females from screened participants were excluded because they failed to fulfill the inclusion criteria. Thus, 222 females were included in the study.

**Table I:** Demographic data for females with probable CTS and non-CTS and possible associated risk factors.

Variable	With probable CTS (n = 76)	Non-CTS (n = 146)	P value
Age, mean ± SD (year)	29.68 (9.98)	27.51 (9.85)	0.000
BMI, mean ± SD (kg/m^2^)	25.09 (4.65)	23.97 (4.40)	0.000
Job, n (%)			0.001
Academic	16 (21.1)	36 (24.7)	
Employees	24 (31.6)	16 (11.0)	
Undergraduates	36 (47.4)	94 (64.4)	
hours of using touchscreen /day, mean ± SD h/d	8.58 (4.94)	7.77 (3.63)	0.000
years of using touchscreen, mean ± SD	8.61 (2.09)	8.09 (2.97)	0.000
Predominance, n (%)			0.000
Right	68 (89.5)	138 (94.5)	
Left	8 (10.5)	8 (5.5)	
Used Finger, n (%)			NS
Thumb	36 (47.4)	56 (38.4)	
Both Thumbs	26 (34.2)	58 (39.7)	
Index	14 (18.4)	32 (21.9)	

The average values of the ROM of the wrist for the tested groups (non-CTS and the probable CTS cohorts) are displayed in Table-II. The MANOVA was computed for the wrist range of motion in non-CTS and probable CTS cohorts at the university female touchscreen users (academic members, employees, and undergraduates).

**Table II:** Wrist range of motion of academic members, employees, and undergraduates of females with probable CTS and non-CTS.

Wrist ROM	Academic members			Employees			Undergraduates		

	Probable CTS, n = 16	Non-CTS, n = 36	P value	Probable CTS, n = 24	Non-CTS, n = 16	P value	Probable CTS, n = 36	Non-CTS, n = 94	P value

	(Mean ± SD)	(Mean ± SD)		(Mean ± SD)	(Mean ± SD)		(Mean ± SD)	(Mean ± SD)	
Flexion °*	70.30 ± 7.74	77.47 ± 8.59	0.001	73.14 ± 9.08	77.90 ± 7.62	0.001	74.06 ± 6.02	80.54 ± 6.31	0.001
Extension °	69.30 ± 9.55	75.89 ± 7.53	0.127	65.24 ± 6.59	68.62 ± 5.29	0.127	72.69 ± 9.08	75.06 ± 7.00	0.127
Radial deviation °	32.15 ± 5.30	39.54 ± 7.73	0.125	21.93 ± 5.56	27.47 ± 8.35	0.125	28.26 ± 8.81	28.42 ± 7.74	0.125
Ulnar deviation °	59.10 ± 6.38	62.50 ± 8.64	0.100	58.71 ± 9.64	65.70 ± 2.69	0.100	62.41 ± 6.24	63.13 ± 8.66	0.100

SD: standard deviation,

* Significant, p < 0.05.

There was no interaction effect between the examined cohorts (females with probable CTS and non-CTS) and the sub-classifications (academic members, employees, and undergraduates) of the touchscreen users. Furthermore, Tukey’s test revealed a significant decrease in the wrist flexion ROM of the probable CTS group (*P*=0.001), as shown in Table-II. In contrast, there was no significant difference between the non-CTS and probable CTS groups for the wrist ROM of extension, radial and ulnar deviation range (*P* > 0.05); however, the ROM is lower in probable CTS.

A number of CTS risk factors appeared to be significantly associated with CTS symptoms. These factors include age, BMI, job, the predominant hand, years and hours of using touchscreen/day. However, no significant associations were found between CTS symptoms and the finger used as shown in Table-I.

## DISCUSSION

This is a novel research exploring the prevalence of CTS symptoms among a cohort of touchscreen users at Majmaah University. The results of the present study found a high prevalence for CTS symptom; more than 34% of the participants reported a probable CTS. This observational study was in concur with Woo^13^ who indicated that the longer computer use was associated with more prevalent wrist/hand discomfort. However, the previous study focuses only on undergraduate touchscreen users that included; laptop and desktop computer users. The prevalence of CTS symptoms in the present study was higher than that suggested by previous studies.^14^,^15^ The outcome differences could be related to the differences in the study populations (male rather than female participants in the current study), different occupations studied, or in the criteria for specifying pain or symptoms.

The prevalence of CTS symptoms may be linked to keeping faulty posture while using touchscreen devices. A number of previous studies stated that people who used to type on cell phone devices with a faulty posture assumed a flexed neck and non-neutral wrist postures.^16^-^18^ Such faulty posture could increase pressure in the carpal tunnel and may progress to CTS. The essence of the typing process over the touchscreen requires repetitive finger movements e.g. clicking, scrolling, swiping, tapping, and pressing buttons. This also could influence fingertip forces, tendon excursion, and muscular effort.^19^

### Wrist Range of Motion

The present study results highlighted that the flexion range of the wrist was significantly decreased in probable CTS females. Additionally, the other wrist movements tended to have lower ROM compared to the non-CTS group. This may return to the extreme wrist movements affect the carpal tunnel pressure. Consequently, females with probable CTS trying to reduce the carpal tunnel pressure and the subsequent pain by limiting the ROM to a certain pain-free range. Many studies supported this idea, as they proposed a strong relation between the pressure along the median nerve inside the carpal canal and wrist position (i.e. deviation from neutral wrist position will lead to increase the pressure over the median nerve which in turn results in aggravation of CTS symptoms).^20^, ^21^ To the best of our knowledge, the posture of wrist and fingers is critical while using the touchscreen devices either for effective performance or for the distribution of the hand pressure. Cha^22^ explained that the peak pressure changed significantly in 15, 30, and 45 degrees’ wrist extension and this rely greatly on the degree of finger flexion. In addition, a variety of thumb movements are required either to reach a specific mobile key or to touch different places over the touchscreen.^23^ More specifically, the highest thumb motor performance is achieved when using the thumb away from its extremes range of motion.

The present study findings suggested that there was a significant correlation between age and CTS symptoms (p = 0.000). This finding appears to be in concur with the findings of Anton, Rosecrance^15^ and Bodofsky^24^, who reported that the CTS severity increased with age. This association may be explained by the vascular abnormalities and axonal loss linked to the aging process that could increase the compression vulnerability of the peripheral nerve, particularly the median nerve, irrespective the duration of symptom.^25^ The results suggested that the increase in the BMI potentially increase the risk of CTS symptoms. Several studies have revealed the association between obesity and CTS.^15^,^26^ The significant association between CTS symptoms and BMI may be due to increased fat deposition which may decrease the carpal tunnel space or due to increase the hydrostatic pressure in the carpal tunnel in obese persons.^27^

The significant associations between CTS symptoms and predominant hand, duration of using touchscreen observed in the study population of the current study may be illustrated by assuming a prolonged poor static posture alongside a repetitive use of the wrist and thumb during touchscreen operation. For example, smartphone may negatively influence the muscular and nervous tissue in the hand^28^, change carpal tunnel pressure^22^ and as a result wrist and hand pain may be developed, particularly on the most frequently used side.^29^ Additionally, using small-screen hand-held devices needed to be hold with certain wrist angles. Such position achieved with highly repetitive movements of the fingers and thumbs in pressing buttons may consequently affect the force generating capacity of the finger muscles.^19^ Moreover, repetitive bending/twisting of the hands/wrists is considered an important risk factor for work-related carpal tunnel syndrome.^26^ The present findings indicate that the higher percent of probable CTS was found in female undergraduates (47.4%). Such finding was in line with Mohammad^30^ However, a direct comparison cannot be made due to studying the risk factors for neck pain rather than hand/wrist pain.

### Limitation of the study

The study was limited to female group only because there are important anatomical and anthropometrical differences between genders that may lead to discrepancy in the results. In addition, it was limited to active workers in certain occupations.

## CONCLUSION

Female touchscreen users at Majmaah University seemed to be at a high-risk cohort for developing CTS, especially undergraduates. Furthermore, the wrist flexion ROM could be a valuable indicator for anticipating variations in the wrist posture after prolong touchscreen use. In addition, the job nature, age, BMI, predominant hand years and hours of using touchscreen/day were found to be risk factors for CTS symptoms.
